# Household Transmission of Influenza A(H1N1)pdm09 in the Pandemic and Post-Pandemic Seasons

**DOI:** 10.1371/journal.pone.0108485

**Published:** 2014-09-25

**Authors:** Itziar Casado, Iván Martínez-Baz, Rosana Burgui, Fátima Irisarri, Maite Arriazu, Fernando Elía, Ana Navascués, Carmen Ezpeleta, Pablo Aldaz, Jesús Castilla, the Primary Health Care Sentinel Network of Navarra

**Affiliations:** 1 Complejo Hospitalario de Navarra, Pamplona, Spain; 2 Instituto de Salud Pública de Navarra, Pamplona, Spain; 3 CIBER Epidemiología y Salud Pública (CIBERESP), Madrid, Spain; 4 Dirección de Atención Primaria, Servicio Navarro de Salud, Pamplona, Spain; 5 Centro de Salud de San Juan, Servicio Navarro de Salud, Pamplona, Spain; Arizona State University, United States of America

## Abstract

**Background:**

The transmission of influenza viruses occurs person to person and is facilitated by contacts within enclosed environments such as households. The aim of this study was to evaluate secondary attack rates and factors associated with household transmission of laboratory-confirmed influenza A(H1N1)pdm09 in the pandemic and post-pandemic seasons.

**Methods:**

During the 2009–2010 and 2010–2011 influenza seasons, 76 sentinel physicians in Navarra, Spain, took nasopharyngeal and pharyngeal swabs from patients diagnosed with influenza-like illness. A trained nurse telephoned households of those patients who were laboratory-confirmed for influenza A(H1N1)pdm09 to ask about the symptoms, risk factors and vaccination status of each household member.

**Results:**

In the 405 households with a patient laboratory-confirmed for influenza A(H1N1)pdm09, 977 susceptible contacts were identified; 16% of them (95% CI 14–19%) presented influenza-like illness and were considered as secondary cases. The secondary attack rate was 14% in 2009–2010 and 19% in the 2010–2011 season (p = 0.049), an increase that mainly affected persons with major chronic conditions. In the multivariate logistic regression analysis, the risk of being a secondary case was higher in the 2010–2011 season than in the 2009–2010 season (adjusted odds ratio: 1.72; 95% CI 1.17–2.54), and in children under 5 years, with a decreasing risk in older contacts. Influenza vaccination was associated with lesser incidence of influenza-like illness near to statistical significance (adjusted odds ratio: 0.29; 95% CI 0.08–1.03).

**Conclusion:**

The secondary attack rate in households was higher in the second season than in the first pandemic season. Children had a greater risk of infection. Preventive measures should be maintained in the second pandemic season, especially in high-risk persons.

## Introduction

Influenza transmission in human beings occurs primarily via the droplet and contact routes [1]. During the 2009–2010 season a new influenza A(H1N1)pdm09 virus emerged, and this virus continued to circulate over the next influenza season. Influenza A(H1N1)pdm09 virus was highly transmissible in schools and households [2]. The World Health Organization (WHO) estimated a secondary household attack rate of the influenza A(H1N1)pdm09 virus of between 22% and 33%, considerably higher than the secondary attack rate for seasonal influenza, which is between 5% and 15% [3].

Several studies had evaluated household transmission of seasonal influenza [4]–[6]. The emergence of a new influenza virus during the 2009–2010 season sparked interest in studying the transmission during the pandemic [7]–[9]. Understanding household transmission is of particular importance in designing more effective preventive strategies.

The onset and spread of the infection within households according to characteristics of the household and the household members during the pandemic season 2009–2010 have been studied previously [10]–[12]. Household transmission in the second season of pandemic virus circulation was studied in the follow-up analysis of 328 households with children in Australia [13]. These studies have focused on influenza transmission during a single season, but to date household transmission in the two seasons of influenza A(H1N1)pdm09 virus circulation has not been compared.

The aim of this study was to analyse household transmission of influenza A(H1N1)pdm09 virus during the first two seasons of pandemic virus circulation, quantifying the incidence of secondary cases generated among the household contacts of laboratory-confirmed index cases.

## Materials and Methods

### Ethics Statement

The index cases were detected as part of the influenza sentinel surveillance with documented oral consent, following the procedure established in the Spanish Influenza Sentinel Surveillance System (http://vgripe.isciii.es/gripe/inicio.do). The protocol with the specific activities of the present study was reviewed and approved by the Ethical Committee of the IMIM-Hospital del Mar Research Institute of Barcelona, Spain. The database for this study included only anonymous data.

### Study population and data collection

The study was conducted in Navarra, Spain, during influenza seasons 2009–2010 and 2010–2011. The primary care sentinel surveillance network included 76 physicians and paediatricians and covered 16% of the population. Network members took nasopharyngeal and pharyngeal swabs after obtaining verbal informed consent from all their patients diagnosed with influenza-like illness whose symptoms had begun within the previous 5 days. Swabs were processed by reverse transcription polymerase chain reaction assay, and samples positive for influenza A(H1N1)pdm09, A(H3N2) and B viruses were identified [14].

A public health nurse telephoned the households of patients who had had laboratory-confirmed pandemic influenza A(H1N1)pdm09 in each season. The surveys were conducted between March and May 2010 in the 2009–2010 season and in February 2011 in the 2010–2011 season. When no response was received, the calls were repeated five times on different days and times. The interview was conducted using a structured questionnaire. For each household, an attempt was made to talk to the adult who was primarily responsible for health issues in the home, usually the mother or father. When possible, other adults in the household were also interviewed. Detailed information was obtained about the index case and the other persons living in the same household with regard to sociodemographic data, medical history, influenza vaccination in the current season, and influenza symptoms. Dates of symptom onset in the household contacts were asked taking as reference the date of symptom onset in the index case, which was obtained from clinical records. A person was considered vaccinated if he/she had received pandemic vaccine in the 2009–2010 season, or seasonal vaccine in the 2010–2011 season, as both contained a vaccine strain against pandemic virus.

### Definitions

*Influenza-like illness* (ILI) was defined as fever and either cough or sore throat in the absence of other diagnoses.

*Index cases* were ILI patients laboratory-confirmed for influenza A(H1N1)pdm09 during the 2009–2010 and 2010–2011 seasons in Navarra, who were surveyed and who had one or more household contacts.

*Household contacts* were persons who had made at least one overnight stay in the house within 7 days before or after the onset of symptoms in the index case. Household contacts who displayed symptoms before the onset of symptoms in the index case were excluded from the study.

*Secondary cases* were susceptible household contacts who had ILI within 7 days from the onset of symptoms in the index case.

*Secondary attack rate* (*SAR*) was calculated as the number of secondary cases divided by the number of susceptible household contacts.

*Transmission rate in households* was defined as the number of households with at least one secondary case divided by the number of households with at least one susceptible contact.

*Serial interval* (interval between infections) was calculated as the number of days between the onset of symptoms in the secondary case and the onset of symptoms for that households index case patient.

### Statistical Analysis

Mean and median serial intervals were calculated based on the date of onset of symptoms in the index and secondary cases. Student’s t test was used to compare means.

A descriptive analysis of the main factors associated with influenza household transmission in each season was carried out. For this purpose, individuals were stratified by age in four groups (0–4, 5–17, 18–49 and ≥50 years) and according to size of municipality of residence in two groups, urban and rural (over or under 10,000 inhabitants). Furthermore, households were classified according to the number of household members (2–3, 4–5 and ≥6) and its size (<80, 80–140 and >140 m^2^). Univariate logistic regression analysis was performed to assess the association between the main characteristics and risk factors of the household contacts and the occurrence of secondary cases in each influenza season.

The occurrence of ILI in household contacts was analysed using a multivariate logistic regression analysis adjusted for the age of contacts, sex, major chronic conditions, vaccination status, sharing a bedroom with the index case, number of household members, rural or urban municipality of residence, age of the index case and influenza season. The measure of association was the odds ratio (OR) with its 95% confidence interval (95% CI).

## Results

### Study population

**Figure 1:**
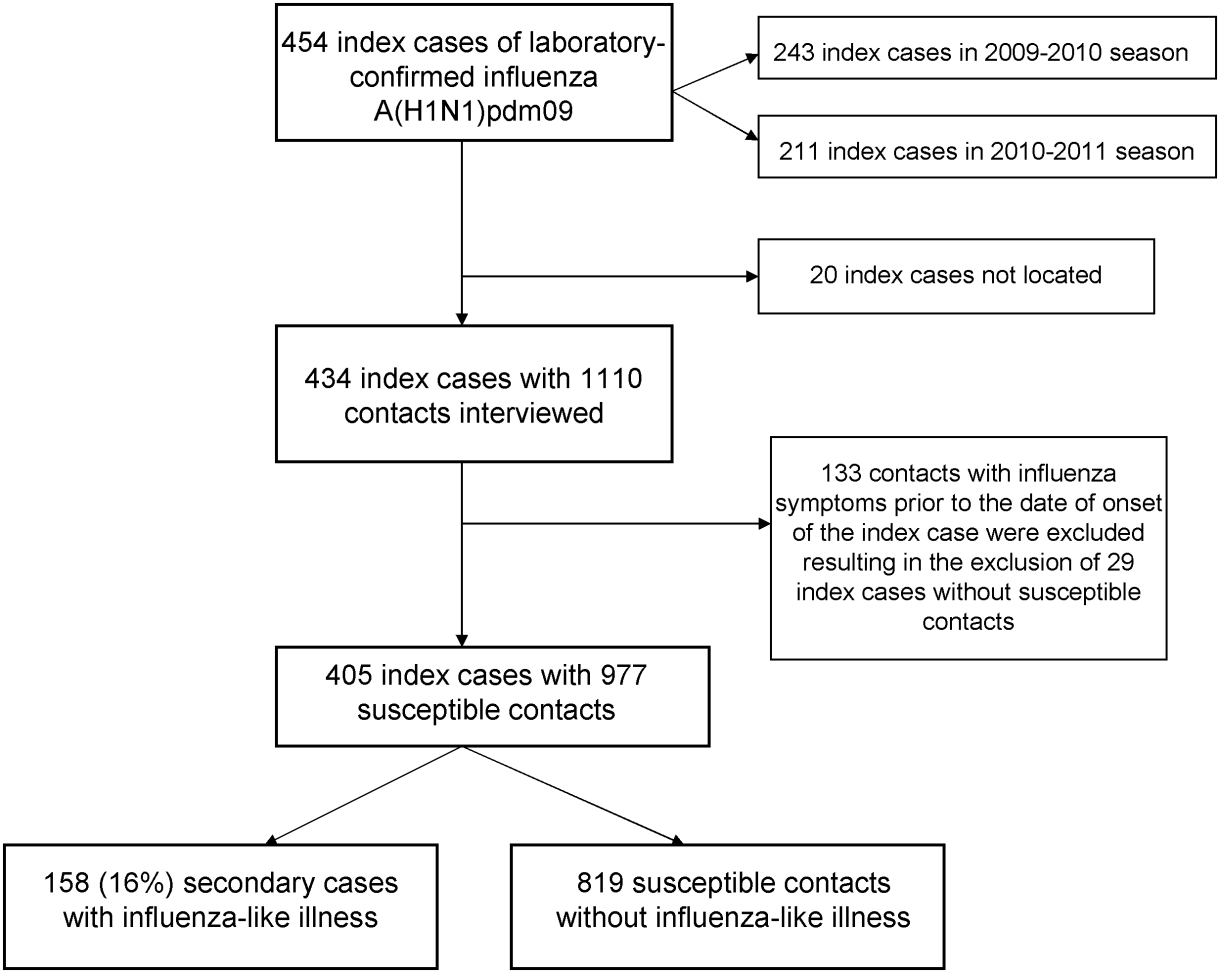
Flow chart of households of index cases with laboratory-confirmed influenza A(H1N1)pdm2009 and their contacts in the study of the 2009–2010 and 2010–2011 influenza seasons in Navarra, Spain.

A total of 454 influenza A(H1N1)pdm09 cases were confirmed in the two study seasons, and 434 of them were contacted, resulting in a response rate of 96%. Symptom onset in the index cases included in the study in the 2009–2010 season occurred between 20 October and 17 November 2009, and in the 2010–2011 season between 3 January and 25 February 2011. The average time between symptom onset and interview was 20 weeks in the 2009–2010 season and 5 weeks in the 2010–2011 season. In the households included in the study 1110 contacts were identified, 133 of which were excluded as they had influenza symptoms prior to the date of onset of symptoms in their index case. Twenty-nine households were excluded because the index case had no susceptible household contacts. Thus, the study focused on the remaining 405 households, 223 from the 2009–2010 season and 182 from the 2010–2011 season, and 977 household contacts. A total of 158 (16%) of the susceptible contacts were secondary cases with ILI (Figure 1).

Of the susceptible contacts, 50% were 18 to 49 years old, 21% had shared a bedroom with the index case, and 72% lived with 4 or more household members. Only 5% of the susceptible contacts had been vaccinated in the corresponding influenza season. The proportion of susceptible contacts under 18 years of age was higher in the 2009–2010 season (30%) than in the 2010–2011 season (25%, p = 0.089).

### Secondary attack rate and serial intervals

Overall, 158 secondary cases occurred among the 977 household contacts, giving a SAR of 16% (95% CI 14–19%). The SAR was higher in the 2010–2011 season than in the 2009–2010 season (19% and 14%, respectively, p = 0.049).

The mean serial interval was 3.7 days (95% CI 3.5–4.0) and the median serial interval was 4 days (interquartile range 2–5). The mean serial interval was longer in the 2009–2010 season (4.0 days) than in the 2010–2011 season (3.4 days; p = 0.037) (Table 1).

**Table 1:** Secondary attack rate, household transmission rate and serial interval of influenza A(H1N1)pdm09 in Navarra in the 2009–2010 and 2010–2011 seasons.

	Total	2009–2010 season	2010–2011 season
**Transmission to susceptible contacts**			
Susceptible contacts (n)	977	594	383
Secondary cases with ILI (n)	158	85	73
Secondary attack rate, % (CI 95%)	16 (14–19)	14 (12–17)	19 (17–22)
**Transmission by household**			
Households (n)	405	223	182
Households with secondary cases with ILI (n)	119	63	56
Household transmission rate, % (CI 95%)	29 (25–34)	28 (22–34)	31 (24–38)
**Serial interval (days)**			
Mean (CI 95%)	3.7 (3.5–4.0)	4.0 (3.6–4.4)	3.4 (3.0–3.8)
Median (interquartile range)	4 (2–5)	4 (3–5)	3 (2–5)

ILI: influenza-like illness.

CI: confidence interval.

The SAR did not differ with regard to sex of the household contacts in either influenza season. In the 2009–2010 season, a decreasing trend of the SAR was observed with increasing age of the contacts: 32% in children under 5 years, 26% in persons between 5 and 17 years old, 10% in persons between 18 and 49 years, and 7% in persons age 50 or older. However, in the 2010–2011 season children under 5 years had a higher SAR (41%) than all other age groups (Table 2).

**Table 2:** Incidence of influenza-like illness and associated factors in household contacts of index cases with laboratory-confirmed influenza in Navarra in the 2009–2010 and 2010–2011 seasons.

	2009–2010 season				2010–2011 season			
	TotalN	With ILIN (%)	Crude oddsratio (95% CI)	P value	TotalN	With ILIN (%)	Crude oddsratio (95% CI)	P value
**Age of the contact (years)**								
0–4	37	12 (32)	1		29	12 (41)	1	
5–17	141	36 (26)	0.71 (0.33–1.57)	0.401	65	11 (17)	0.29 (0.11–0.77)	0.013
18–49	311	30 (10)	0.22 (0.10–0.49)	<0.001	180	39 (22)	0.39 (0.17–0.89)	0.025
≥50	103	7 (7)	0.15 (0.05–0.43)	<0.001	109	11 (10)	0.16 (0.06–0.42)	<0.001
**Sex**								
Male	301	41 (14)	1		187	35 (19)	1	
Female	293	44 (15)	1.12 (0.71–1.77)	0.627	196	38 (19)	1.04 (0.63–1.74)	0.867
**Major chronic conditions**								
No	517	78 (15)	1		320	57 (18)	1	
Yes	77	7 (9)	0.56 (0.25–1.27)	0.166	63	16 (25)	1.57 (0.83–2.97)	0.164
**Smoker**								
No	505	74 (15)	1		324	64 (20)	1	
Yes	89	11 (12)	0.82 (0.42–1.62)	0.569	59	9 (15)	0.73 (0.34–1.56)	0.420
**Vaccination status**								
No	583	85 (15)	1		346	70 (20)	1	
Yes	11	0 (0)	NA	0.379	37	3 (8)	0.35 (0.10–1.17)	0.087
**Shared bedroom with index case**								
No	475	64 (14)	1		295	51 (17)	1	
Yes	119	21 (18)	1.38 (0.80–2.36)	0.246	88	22 (25)	1.60 (0.90–2.82)	0.108
**Number of household members**								
2–3	110	17 (16)	1		166	25 (15)	1	
4–5	398	60 (15)	0.97 (0.54–1.74)	0.922	195	46 (24)	1.74 (1.02–2.98)	0.044
≥6	86	8 (9)	0.56 (0.23–1.37)	0.204	22	2 (9)	0.56 (0.12–2.57)	0.459
**Household size**								
<80 m^2^	104	14 (14)	1		99	22 (22)	1	
80–140 m^2^	382	54 (14)	1.06 (0.56–1.99)	0.860	252	50 (20)	0.87 (0.49–1.53)	0.619
>140 m^2^	95	14 (15)	1.11 (0.50–2.47)	0.796	32	1 (3)	0.11 (0.02–0.87)	0.037
**Residence**								
Rural	151	28 (19)	1		32	9 (28)	1	
Urban	443	57 (13)	0.65 (0.40–1.07)	0.087	351	64 (18)	0.57 (0.25–1.29)	0.177
**Age of the index cases (years)**								
0–4	26	3 (12)	1		13	4 (31)	1	
5–17	290	41 (14)	1.26 (0.36–4.40)	0.714	84	18 (21)	0.61 (0.17–2.23)	0.457
18–49	239	39 (16)	1.50 (0.43–5.22)	0.529	252	47 (19)	0.52 (0.15–1.75)	0.287
≥50	39	2 (5)	0.41 (0.06–2.67)	0.354	33	4 (12)	0.31 (0.06–1.50)	0.145

NA: not available; ILI: influenza-like illness; CI: confidence interval.

In separate analyses for the two seasons, no statistically significant differences were found in the SAR by sex, major chronic conditions, tobacco use or influenza vaccination in the contact, sharing a bedroom with the index case, urban or rural environment, or with the age of the index case. In the 2010–2011 season, the SAR was higher among those contacts living in 4- or 5-member households than in those with only 2 or 3 members (p = 0.044), whereas living in households with 6 or more members was not associated with an increased SAR (Table 2).

The comparison between the two seasons showed statistically significant increases in the SAR among susceptible contacts with major chronic conditions (9% to 25%, p = 0.010) and among those contacts living in 4- or 5-member households (15% to 24%, p = 0.011).

The household transmission rate for both seasons averaged 29%, with no statistically significant difference between the 2010–2011 season (31%) and the 2009–2010 season (28%, p = 0.580).

### Factors associated with household transmission

The multivariate regression analysis showed that household transmission of influenza was more likely in the 2010–2011 season than in the 2009–2010 season (OR: 1.72; 95% CI 1.17–2.54).

Regarding the age of household contacts, the transmission risk was highest in children under 5 years old, and this risk decreased with the age of the contacts, reaching an OR of 0.14 (95% CI 0.07–0.30) in those aged 50 years and more. However, the age of the index case was not associated with differences in the risk of household transmission.

There were no differences in the probability of transmission of the influenza virus by sex or major chronic conditions of the contacts. Influenza vaccination was associated with a lower incidence of ILI in the household contacts near to statistical significance (OR: 0.29; 95% CI 0.08–1.03).

Household contacts who shared a bedroom with the index case had a higher risk of presenting ILI (OR: 1.99; 95% CI 1.27–3.11), and people living in households of 6 or more members showed a lower risk than those living in households of 2 or 3 members (OR: 0.40; 95% CI 0.18–0.90). Living in an urban versus rural area was also protective (OR: 0.61; 95% CI 0.39–0.96) (Table 3).

**Table 3:** Household transmission of laboratory-confirmed influenza A(H1N1)pdm09 by characteristics of the contact and of the household, and by age group of the index case in Navarra in the joint analysis of the 2009–2010 and 2010–2011 seasons.

	Number of contacts		Adjusted analysis^b^	
	Total^a^	With ILI (%)	Odds ratio (95% CI)	P value
**Age of the contact (years)**				
0–4	66	24 (36)	1	
5–17	206	47 (23)	0.51 (0.27–0.95)	0.033
18–49	491	69 (14)	0.22 (0.12–0.41)	<0.001
≥50	211	18 (9)	0.14 (0.07–0.30)	<0.001
**Sex**				
Male	487	76 (16)	1	
Female	487	82 (17)	1.18 (0.83–1.69)	0.361
**Major chronic conditions**				
No	834	135 (16)	1	
Yes	140	23 (16)	1.60 (0.94–2.73)	0.086
**Vaccination status**				
No	926	155 (17)	1	
Yes	48	3 (6)	0.29 (0.08–1.03)	0.055
**Shared bedroom with index case**				
No	768	115 (15)	1	
Yes	206	43 (21)	1.99 (1.27–3.11)	0.003
**Number of household members**				
2–3	275	42 (15)	1	
4–5	592	106 (18)	1.06 (0.68–1.65)	0.811
≥6	107	10 (9)	0.40 (0.18–0.90)	0.026
**Residence**				
Rural	182	37 (20)	1	
Urban	792	121 (15)	0.61 (0.39–0.96)	0.031
**Age of the index cases (years)**				
0–4	39	7 (18)	1	
5–17	374	59 (16)	0.93 (0.38–2.28)	0.870
18–49	489	86 (18)	0.80 (0.33–1.97)	0.628
≥50	72	6 (8)	0.50 (0.14–1.78)	0.287
**Season**				
2009–2010	592	85 (14)	1	
2010–2011	382	73 (19)	1.72 (1.17–2.54)	0.006

ILI: influenza-like illness.

CI: confidence interval.

a Three contacts were excluded due to missing values in variables included in the analysis.

b Logistic regression analysis adjusted for variables in the table.

The analysis was repeated, first excluding household contacts that had been vaccinated against flu, and second excluding household contacts younger than 18 years old, and the estimates obtained were similar.

## Discussion

This is the only study that has evaluated influenza transmission in households in the first two seasons following the introduction of the pandemic influenza virus A(H1N1)pdm09. The average SAR was 16%, and 29% of households with an index case had at least one secondary case. Interestingly, the SAR in households was higher in the second wave, an increase that mainly affected persons with major chronic conditions.

The SAR detected for pandemic influenza in households was 16%, lower than the 22–33% rate initially estimated by WHO [3]. A recent systematic review of studies of household transmission during the 2009 pandemic reported SARs ranging from 3% to 38%, and suggested that part of this variability may be due to the different designs used in the studies included in the review [15]. A study carried out in Spain with 2039 susceptible household contacts found a SAR of 11% [16], lower than observed in our results. A study by Carcione et al. [12], developed with a similar methodology in 595 Australian households in the 2009–2010 season, found a SAR of 15%, close to the one observed in our study.

Children are more likely to become infected than adults, as has been described in other studies [11], [12], [17], and this likelihood decreases with age [18]. A systematic review published by Glatman-Freedman et al. analysed the SAR in children and their contacts during the pandemic influenza season, and concluded that children have a significantly higher SAR compared to adults, both in laboratory-confirmed and clinical cases, and in various settings and locations around the world [19]. This increased susceptibility of infection may be due to the fact that children have more physical contact with others than do adults and are less likely to be protected by prior immunity [18].

The likelihood of transmission was higher in contacts who shared a bedroom with the index case, as has been reported elsewhere [8]. Transmission was also more likely in rural households. Living in a house with 6 or more members reduced the probability of infection, as was also noted in a study in the United States [17]. In households with few members, contacts between any two of them may be closer than in households with more members. However, this reduced probability of infection was not consistently observed during the 2009 pandemic, as was noted in a systematic review of studies on household transmission [15]. We did not observe a higher SAR when the index cases were young children; this result is similar to the findings of some previous studies [10], [17], but contrasts with what other authors have reported [12], [20].

In our study, previous vaccination against influenza A(H1N1)pdm09 virus was protective against household transmission of influenza. This is consistent with the results of a study carried out in 1614 households in Japan, which observed a protective effect of pandemic influenza vaccine in children [21].

Transmission was higher in the 2010–2011 season than in the 2009–2010 season (19% vs. 14%). This might be due to several reasons. The pandemic wave peaked in October in the 2009–2010 season [14], whereas the peak in the 2010–2011 season occurred in January [22]. This could partly explain the higher transmission in the second season since influenza virus transmission is favoured by a cold and dry environment [23]. During the pandemic season public health authorities stressed the importance of adopting hygienic measures, hence the population might have been more conscious about preventive messages than in the subsequent season. A household-based study conducted during pandemic season 2009–2010 in Germany observed that 49% of index cases and 55% of household contacts cleaned their hands more often in the week after symptom onset in the index case, whereas 30% in both groups did so during the 2008–2009 pre-pandemic season. Moreover, in the pre-pandemic season only 18% of the index patients cleaned their hands regularly after coughing or sneezing, a proportion that rose to 48% in the pandemic season [24]. The increase in the SAR among persons with major chronic conditions can be explained by a certain relaxation in the second pandemic season of the intensive preventive measures aimed at high risk persons in the first pandemic season.

The mean serial interval in our study was 3.72 days, within the range observed in the systematic review by Lau et al. [15]. The serial interval was longer in the 2009–2010 season than in the 2010–2011 season, which is consistent with the fact that higher SARs are associated with shorter serial intervals [25].

The strengths of this study are the large number of households included and the representativeness of the index cases of the region, since they were detected from the Sentinel Network of Primary Care Physicians and Paediatricians of Navarra. Moreover, the study analyses and compares two seasons with circulation of the pandemic A(H1N1)pdm09 virus.

Our study has several limitations. We used a clinical definition for secondary cases, and they were not confirmed by laboratory; however, all of them had had recent contact with a laboratory-confirmed index case, therefore they are highly likely to have had influenza infection. Asymptomatic infections were not considered, which would have increased the SAR. There could be a recall bias, since in some households the period between illness in the index case and application of the questionnaire could have been several weeks. This interval was longer in the pandemic season which, in principle, could affect the comparability between seasons. However, to prevent this potential bias the dates of symptom onset in the household contacts were established taking as reference date of symptom onset in the index case, which was previously registered, and the special situation of the pandemic could also make recall easier. Finally, some secondary cases could have acquired the infection outside the household, as transmission of the influenza A(H1N1)pdm09 virus in school outbreaks has been shown to be quite efficient in children [2], [7], thus over-estimating the SAR. However, the risk of transmission from community sources is very low as compared to the risk of transmission from a laboratory-confirmed index case in the household [26]. A follow-up study of a household cohort in Vietnam that included virus genetic sequencing concluded that 91% of the secondary cases were infected within households rather than from the community [27].

Preventive measures should be applied within households, with special focus on children [19]. Annual vaccination against influenza is the main preventive measure available, and should be complemented with hygienic measures such as washing hands more frequently and covering the mouth when coughing and sneezing. A multicentre study carried out in 36 Spanish hospitals proves the effectiveness of hand washing and the provision of information on influenza prevention in the community in preventing influenza A(H1N1)pdm09 admissions [28]. The measures to prevent influenza transmission in pandemics should be maintained at least until the immediately following season, since the transmission risk in the second wave may be similar to or higher than in the first season.

## Conclusions

The SAR for influenza A(H1N1)pdm09 in households was higher in the second season with circulation of the pandemic virus than in the first pandemic wave. Children had a greater risk of infection. The main measures that we detected to prevent influenza transmission in households were annual influenza vaccination and not sharing a bedroom with symptomatic persons. In response to pandemic influenza, health authorities should consider maintaining control measures in the first two seasons following the detection of a new influenza virus. Particular emphasis should be put on maintaining preventive measures in households with high-risk persons during the second pandemic season.

## Supporting Information

File S1 **Study database.**
(ZIP)
